# The Local Treatment of Acne

**Published:** 1909-12

**Authors:** 


### The Local Treatment of Acne.

Gaucher is credited by the *Journal de Medicine de Paris* for August 28, 1909, with the following contribution to the subject. It is remarked that there are two drugs which give the gest results in the local treatment of acne—namely, resorcin and sulphur. Resorcin sometimes proves irritating, especially when exhibited in the form of an ointment. It is best therefore to employ it in aqueous solution as follows:

℞ Resorcin ....................... gr. xxx
Distilled water................. ℥iii
M.

Sulphur is frequently used in the form of ointment, but it is an objectionable form, as the greasy mass only augments the abnormal condition of the skin. A more agreeable method is to prescribe it in the following lotion:

℞ Precipitated sulphur.............. ʒiss

Purified talcum.................... ʒss

Refined glycerin.................. ℥iss

Rose water........................ ℥iv

Tincture of soap bark............ ʒiiss

M.

Sublimed sulphur should never be used in lotions intended for the treatment of acne, as it is very irritating to the skin. In preparing the lotion the apothecary should be instructed to incorporate the dry ingredients intimately in a mortar, adding the glycerin, and then the rose water, little by little. The tincture of soap bark, which partially emulsifies the mixture, should be added lastly, a few drops at a time, the mixture being well stirred meanwhile.

The lotion is applied at night, after the face has been bathed in water as hot as can be borne. Wash off in the morning with hot water.—*New York Medical Journal.*

